# Does engagement help to reduce insomnia when workers are emotionally exhausted?

**DOI:** 10.1007/s41105-022-00411-7

**Published:** 2022-09-01

**Authors:** Samuel Fernández-Salinero, Gabriela Topa, Juan José Fernández Muñoz

**Affiliations:** 1 Psychology Department, Universidad Rey Juan Carlos, 28933 Madrid, Spain; 2 Department of Social and Organizational Psychology, National Distance Education University (UNED), 28040 Madrid, Spain

**Keywords:** Insomnia, Emotional exhaustion, Work engagement, Vigor, Dedication, Absorption

## Abstract

Insomnia is one of the most common problems, affecting more than 35% of the world’s population. To achieve a better understanding of this problem the focus of this research is to understand how emotional exhaustion at work may lead to insomnia. To help to combat it, we tested a mediation model including engagement factors. The sample was composed of 823 participants. 38.3% (315 subjects) were male and 61.7% (508 subjects) were female. Mean age was 42.65 years old (9.05 = SD). Main results showed that emotional exhaustion is directly and statistically significant related to insomnia. However, different engagement factors showed different weights in buffering this relationship. Emotional exhaustion showed a statistically significant impact on insomnia. Vigor and absorption helped to buffer the impact of emotional exhaustion over insomnia. Our study has some limitations. First, the sample was acquired by not aleatory processes. Another limitation is that our sample was composed of individuals with decision-making capacity. Lastly, our research is a transversal study. Future research should take these limitations into account and conduct longitudinal research with aleatory sampling procedures.

## Introduction

Despite strong correlations have been established between burnout and insomnia [1–3], the ways in which this relationship and the factors that are involved are still unknown.

Previous research has highlighted that insomnia is a very usual phenomenon. Some reviews have shown that more than 60% of the sample met the criteria of insomnia [4]. Moreover, recent papers have stated that burnout has a very important impact on predicting insomnia because of its affection on sleep physiology, prolonging sleep latency and disturbing rapid eye movement (REM) [2, 3, 5]. Both burnout and insomnia have been posed to affect several physiological and psychological variables, such as emotional exhaustion. However, there are differences in how these relationships happen. For example, some scholars have stated that burnout is not associated with insomnia [6], while other researchers have affirmed the relationship between the two variables [7].

As burnout and insomnia are complex phenomena, one of the main objectives of this research is to clarify and expand the relationships between these two variables. Several scholars have stated that insomnia was only related to the emotional exhaustion burnout component in the working population. This is the departure point of this research that will analyze the effect of emotional exhaustion on insomnia.

Related to insomnia, it is defined as the experience of problems to fall asleep, to maintaining sleep, or to early awakening [8]. These symptoms have an impact on daily life such as impaired attention, problems in concentration and hindered work performance, among others. As recent research state, insomnia may appear before a medical or mental disorder appear. Besides, it may persist beyond the clinical syndrome. It has been proved that insomnia is a very frequent disorder affecting more than 35% of the population, and it is maintained even after the initial stressors stop [9]. Moreover, it has been stated that insomnia costs a lot of resources to the health system of developed countries. For example, it has been related to depressive or suicidal symptoms in previous research [8]. Moreover, recent researches have highlighted the relationship between dispositional factors such as personality and anxiety and depression symptoms, being these relationships mediated by insomnia [10].

In the organizational field, insomnia has been related to occupational stress and physical health problems. Some scholars showed the relationships between burnout and insomnia and considered them relatively constant and chronic issues [11]. Moreover, it has been found that insomnia is related to the intention to reduce working hours, intention to change job content and quit employment (Ying-Chen et al., 2010). Having said this, the current research tries to clarify the way in which burnout may conduct to insomnia.

On the other hand, work engagement has been defined as a positive, fulfilling, work-related state of mind, which is characterized by vigor, dedication, and absorption [12, 13]. Several research papers have pointed out the relationships between engagement and positive demand coping strategies, while burnout responses have been related to negative or avoidance coping strategies (Pienaar and Willemse 2008). In this line, recent studies have shown that engagement may work as a protection against insomnia, and people with higher levels of engagement may be likely to have fewer insomnia symptoms [14].

Due to this, the present study aims to test the relationship between emotional exhaustion and insomnia, but considering the mediation effect of vigor, dedication, and absorption as engagement factors. We follow previous research suggestions where emotional exhaustion has been demonstrated to be a very interesting predictor of sleeping problems [15]. Besides, previous research has shown that some factors of engagement, such as vigour works as buffers of insomnia, while others show a low effect on preventing this variable [16]. Therefore, one of our aims is to test the impact of individual engagement factors as involved variables in the insomnia process.

## Theoretical framework

Burnout is one of the most important variables in predicting organizational health. It is characterized by emotional exhaustion, cynicism, and impaired personal accomplishment. Maslach et al. [17] defined burnout as the experience of emotional exhaustion related to one’s job, where subjects turn cynical towards the value of their work and begin to feel doubtful about their ability to perform. Specifically, Maslach posed that burnout is a construct composed of three main factors. Emotional exhaustion refers to the feeling of stress and fatigue which derives from work demands that exceed the capacity to cope with. On the other hand, cynicism refers to the feeling of being detached from one’s job or the people with whom one works. Finally, loss of self-efficacy refers to the impairment of the beliefs of one capability to conduct their job.

Reviewing burnout literature, some scholars stated that in the same work conditions some workers may become burned out, and some others not, so, burnout may be seen as a response to stressful events [18]. As stated above, emotional exhaustion is one of the most important components that have been related to the impairment in some health variables such as physical or psychological problems [19]. Besides, it has been demonstrated that the emotional exhaustion component fully mediated the relationship between job demands and mental health in some research [20, 21]. Specifically, it has been found that emotional exhaustion was related to insomnia in previous research [15]. So, the focus of this research is to explore how this relationship between emotional exhaustion and insomnia appears. On the line of this research, some scholars have stated that individuals scoring high in burnout showed more short awakening during sleep and impaired sleep efficiency measured with electroencephalogram [22].

In addition, work engagement is a very interesting and popular topic in organizational psychology research. It is associated with wellbeing and performance [23]. Given its importance and positive outcomes, a huge amount of research has focused on discovering its antecedents and outcomes. The root of this concept is in Kahn [24] research, he defined engagement as that stated were employees feel physically, cognitively and emotionally involved in their work roles, feeling a sense of meaning, safety and availability [25]. After some debates around if this construct was opposite to burnout, [12, 13] refuted this argument and developed an instrument to assess this variable.

The authors developed the Utrecht Work Engagement Scale (UWES) which was used in this research and described in the method section. Work engagement was defined as a state of mind characterized by vigor, dedication, and absorption. Vigor refers to the high energy level and mental resilience that may be felt when working. On the other hand, dedication refers to the feeling of being intensely involved in work tasks and experiencing meaningful and challenging work. Finally, absorption is related to the state of concentration at work, and positive engrossment [25].

Engagement concept has had many critics and questions arising around it. Even, some academics have questioned the existence of the concept itself [26]. However, the author’s perspective of engagement is rooted in the Job Demands-Resources model. Within this optic, job resources are physical, social, or organizational aspects that can help individuals to achieve goals and significant learning. This resource-demands imbalance may lead to poor health outcomes such as depression, turnover intention, sickness, or poor performance among others. Burnout may be a negative outcome of chronic demands, while engagement refers to a positive and self-efficacy-based way to cope with demands.

Within this line, a recent meta-analysis has analyzed the most common outcomes of work engagement programs and has identified four types of interventions: personal resources building interventions, job resources building interventions, leadership training interventions and health-promoting interventions [25].

Due to the potential applicability of work engagement, the current research departs from the idea that those individuals who experience levels of burnout and behave in a more positive and healthy way through work engagement, will be less likely to be affected by insomnia. An important idea of this research is to understand what components of engagement are related to prevent insomnia. Thus, our assumption is that work engagement dimensions (vigor, dedication and absorption) are a mediator between emotional exhaustion and insomnia. Due to this, we purpose the following hypotheses.

H1: Burnout (emotional exhaustion) will be direct and significant related to insomnia.

H1a: The dimension vigor will mediate individually the relationships between burnout (emotional exhaustion) and insomnia.

H1b: The dimension dedication will mediate individually the relationships between burnout (emotional exhaustion) and insomnia.

H1c: The dimension absorption will mediate individually the relationships between burnout (emotional exhaustion) and insomnia.

H2: The dimension vigor in serial with dedication will mediate the relationships between burnout (emotional exhaustion) and insomnia.

H3: The dimension vigor in serial with absorption will mediate the relationships between burnout (emotional exhaustion) and insomnia.

H4: The dimension dedication in serial with absorption will mediate the relationships between burnout (emotional exhaustion) and insomnia.

H5: The dimension vigor in serial with dedication and absorption will mediate the relationships between burnout (emotional exhaustion) and insomnia.

## Methods

### Sample

The sample was composed of 823 participants, 38.30% (315 subjects) were male and 61.70% (508 subjects) were female. Mean age was 42.65 years old (9.05 = SD). Related to the education degree, our sample was composed of 18 individuals (6.70%) who had primary education, 95 individuals who completed bachelor’s (11.50%), 113 individuals (13.70%) who completed a vocational training program, and 597 individuals (72.50%) who had a university degree. Regarding to professional category, our sample was composed by 131 individuals (15.90%) who occupied manager positions, 255 individuals (31%) occupied middle-level management positions, 384 individuals (46.70%) occupied administrative posts and 53 individuals (6.4%) had non-qualified posts.

### Procedure

To conduct our research several Spanish organizations were contacted and invited to fill a work insomnia assessment. The study was approved by the ethical committee for research of the National Distance University (VITCDC2019.9.9). The consulted organizations comprise a wide angle of sectors from primary to services. In all the organizations, all the workers were invited to participate, and we collected data about their hierarchy within the organization.

The sampling procedure was carried out by convenience procedure. Several organizations were contacted and invited to participate. If managers agreed to participate, we asked them to share the link of the questionnaire with their workers. Questionnaire was constructed using the Google Forms application, and it was administered in 2020. Anonymity and confidentiality were guaranteed for avoiding biases. Besides, we informed every participant about the conditions of the research, and the voluntary participation. Since we contacted managers, we do not have information about the response rate. This may be a limitation of the sampling procedure. The requirement for participating in this research was to be currently active. There were no exclusion criteria for participating.

### Measures

Maslach Burnout Inventory-MBI (Maslach et al.). This scale has three dimensions. The first one is emotional exhaustion; this factor is composed by a 7-point Likert-type scale of 5 items, ranged from 0 (never) to 6 (everyday). This questionnaire was originally designed by Gil-Monte [27], and emotional exhaustion showed a Cronbach’s alpha value of 0.85. In our sample, the alpha value was 0.80**.** Examples of the items are (“I feel emotionally exhausted by my work”). This questionnaire is used to measure burnout syndrome, regardless of the occupational characteristics of the sample [28]. Emotional exhaustion has been seen as those feelings of being depleted of one’s emotional and physical resources [29]. On the other hand, depersonalization reflects the worker’s detachment response to the aspects of their job. At last, personal accomplishment refers to the employees’ feelings of being competent and productive at work. Of these three factors, emotional exhaustion has been viewed as the primary manifestation of burnout syndrome [30], and as noted above, it has been related to insomnia in previous research.

Spanish validation of the Athens Insomnia Scale-AIS-[31]. To assess this variable, we used the Spanish validation of the Athens Insomnia Scale This instrument was developed originally by Soldatos et al. [32]. It was composed of 8 Likert-type items ranging from 0 to 3. Lower ratings mean lower intensity. The overall scale reliability was Cronbach’s alpha = 0.89. In our study, Cronbach’s alpha value was 0.82. The first four items evaluate the quantitative difficulties of sleep (“Time it takes you to fall asleep once you are in bed”). Item 5 evaluates sleep qualitatively (“No matter how long you slept”). The three last items evaluate the insomnia daily impact (“Wellbeing feeling during the day”).

Utrecht Work Engagement Scale -UWES-[33]. For assessing this variable, we used the Spanish validation [34]. This instrument is composed by 17 Likert-type items ranging from 0 (“never” to 6 (“always”. Confirmatory factorial analysis showed a three factors structure. First vigor is evaluated by six items, and it is referred to the degree of energy that an individual feels (“In my job I feel full of energy”, “I am strong and vigorous in my job”. Second, dedication is evaluated by five items, and it is referred to the meaning of one’s work (“My job is full of meaning and purpose”; “I am enthusiastic about my job”. Third, absorption is evaluated by six items referred to the degree that an individual feels happily immersed in his job (“Time flies when I am working”; “I am immerse in my job”. The overall alpha values were adequate showing a Cronbach’s alpha = 0.93. The vigor factor showed an alpha = 0.86, the dedication factor showed an alpha = 0.92, and the absorption factor showed an alpha = 0.80. In our sample, the alpha values were: overall = 0.87, vigor = 0.88, dedication = 0.87, and absorption = 0.83.

### Data analysis

A mediation model was applied with process PROCESS 2.16.3 [35]. To develop the statistical analysis, several assumptions were checked, for instance, normality (*p* > 0.05, Kolmogorov test), homoscedasticity (*p* < 0.05, Levene test) and independency of the variables included in the model (Durbin-Watson <2). Thus, descriptive analysis, Pearson correlation and hierarchy regression were included in the results chapter. For the serial mediation model, according to Hayes [35], model 6 was applied with a 95% Confidence Interval (CI) and a bootstrapping of 10.000. Moreover, the significant effect of the variables inside the model has decided with *p*-values <0.01 and Lower Level Confidence Interval (LLCI) and Upper-Level Confidence Interval (ULCI). We evaluated the impact of age in our models and did not include it in the final version because it has not any statistically significant impact.

## Results

Table 1 shows the sociodemographic characteristics of the sample.

**Table 1:** Sample sociodemographic characteristics

Variable	*N*	%	M	SD
Age	823	–	42.65	9.05
Gender				
Male Female	315 508	38.30 61.70		
Education				
Primary Bachelor Vocational training University degree	18 95 113 597	2.30 11.50 13.70 72.50		
Job Rank				
Manager Middle-level management Administrative Non-qualified	131 255 384 53	15.90 31.00 46.70 6.40		

Table 2 shows the descriptive results and correlation index There is a significant relationship between insomnia and emotional exhaustion (*r* = 0.49, *p* < 0.01), and negative relationships with vigor (*r* = −0.60, *p* < 0.01), dedication (*r* = −0.52, *p* < 0.01) and absorption (*r* = −0.29, *p* < 0.01).

**Table 2:** Descriptive results and Pearson correlation index for insomnia, emotional exhaustion, vigor, dedication and absorption

Variable	*α*	M	SD	1	2	3	4	5
Emotional exhaustion	0.80	2.68	1.07	1	–	–	–	–
Insomnia	0.82	2.46	0.92	0.49**	1	–	–	–
Vigor	0.88	3.32	0.94	− 0.60**	− 0.31**	1	–	–
Dedication	0.87	3.58	1.00	− 0.52**	− 0.22**	− 0.83**	1	–
Absorption	0.83	3.37	0.91	− 0.29**	− 0.08*	0.65**	0.73**	1

^*^p < 0.05; **p < 0.01

Moreover, a hierarchy regression model was conducted as well. We found a statistically significant relationship between Burnout (emotional exhaustion) and Insomnia (*B* = 0.40, SE = 0.03, 95% CI [0.33, 0.46], *p* < 0.001); vigor (*B* = −0.18, SE = 0.06, 95% CI [−0.29, −0.06], *p* < 0.001) and absorption (*B* = 0.10, SE = 0.05, 95% CI [0.01, 19], *p* < 0.001). The overall model was significant (*R*^2^ = 0.25; MSE = 0.64, *p* < 0.001). In Table 3 below specific regression data are shown.

**Table 3:** Hierarchy regression analysis to determine predictor of insomnia

Variable	B	SE	*p*	Sꞵ	LLCI	ULCI
Exhaustion	0.40	0.03	0.00	0.46	0.33	0.46
Vigor	− 0.18	0.06	0.00	−0.18	− 0.29	− 0.06
Dedication	0.09	0.06	0.12	0.09	− 0.02	0.20
Absorption	0.10	0.05	0.02	0.10	0.01	0.19

*SB* Standardized beta, *SE* Standard error, *LLCI* Lower limits confidence interval, *ULCI* Upper limits confidence interval

Once the regression model was conducted, we evaluated the possibilities of simple mediation models and mediation in serial to check which components of engagements (vigor, dedication and absorption) could mediate the effect of emotional exhaustion on insomnia. Table 4 shows the results of the simple and sin serial mediation model (model 6).

**Table 4:** Mediation model between emotional exhaustion in serial with vigor, dedication and absorption on insomnia

Model	B	SE	LLCI	ULCI
Model 1 (Exh > Vig > Insom)	0.09	0.03	0.03	0.16
Model 2 (Exh > Ded > Insom)	− 0.00	0.00	− 0.01	0.00
Model 3 (Exh > Abs > Insom)	0.02	0.00	0.01	0.03
Model 4 (Exh > Vig > Ded > Insom)	− 0.04	0.03	− 0.10	0.01
Model 5 (Exh > Vig > Abs > Insom)	− 0.01	0.01	− 0.03	− 0.01
Model 6 (Exh > Ded > Abs > Insom)	− 0.00	0.00	− 0.01	0.00
Model 7 (Exh > Vig > Ded > Abs > Insom)	− 0.03	0.01	− 0.05	− 0.01

*Exh* Exhaustion, *Vig* Vigor, *Ded* Dedication, *Abs* Absorption, *Insom* Insomnia

**Fig. 1:**
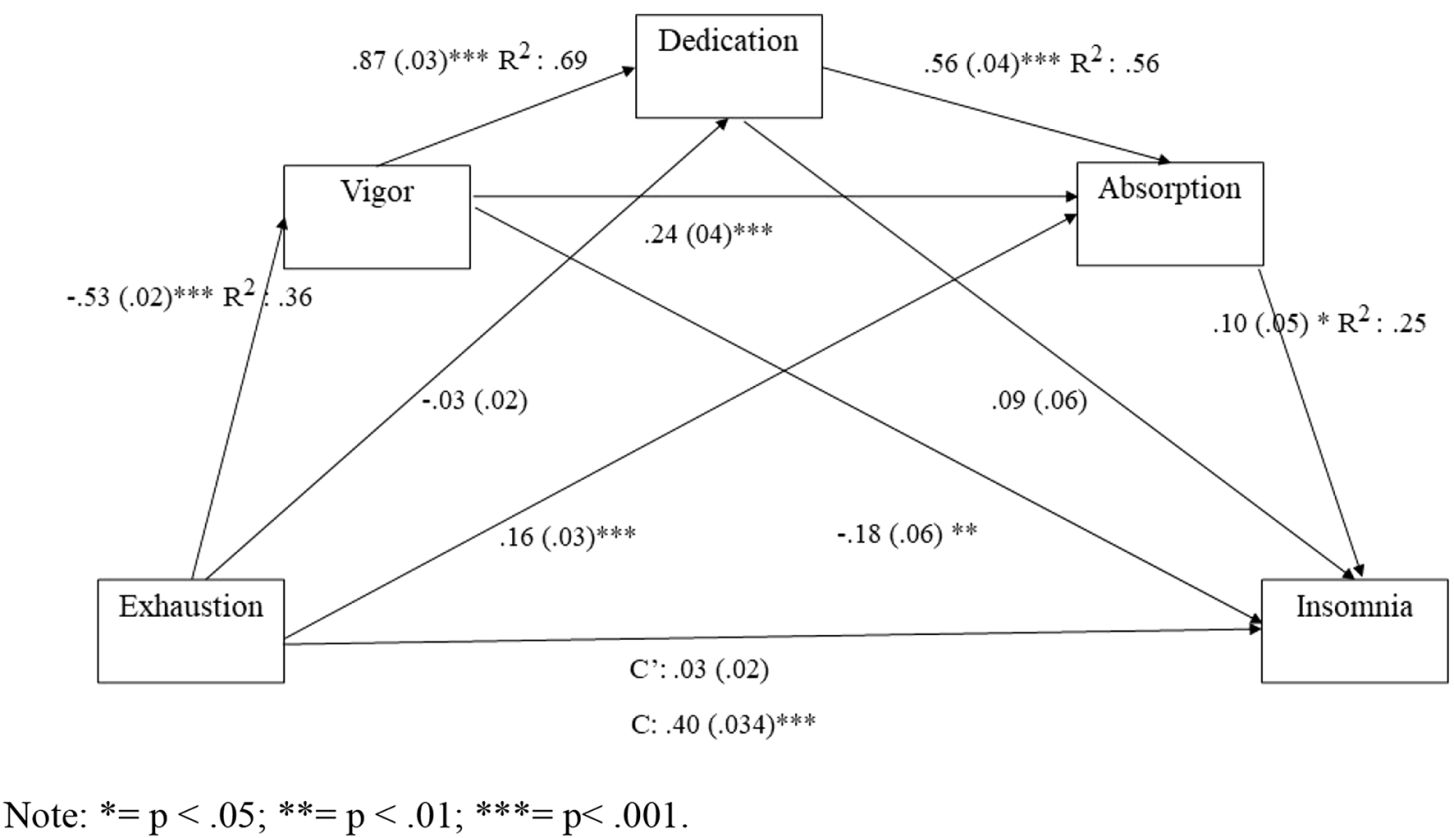
Serial mediation model 6 linking emotional exhaustion and insomnia (*n* = 823) *p < 0.05; **p < 0.01; ***p < 0.001. Serial mediation model 6 linking emotional exhaustion and Insomnia (*n*=823). Standardized effects are presented outside the parentheses with bootstrapped SEs in the parentheses. C’=indirect effect of emotional exhaustion on insomnia; C=direct effect of emotional exhaustion on insomnia

As we can see, the single mediation model is only supported with vigor and absorption, and it tends to buffer the direct impact of burnout over insomnia, but not to revert it. Dedication did not show a relationship to insomnia, as we could appreciate in the direct regression model, but since it is negatively related to burnout and insomnia, we decide to test its influence in the different mediation models. Related to the step mediation models, only model 5 (Burnout→Vigor→ Absorption→ Insomnia) and model 7 (Burnout→ Vigor→ Dedication→ Absorption→ Insomnia) are significant. The best fit model is model 7 which included all the factors of work engagement and achieve to revert the burnout effects over insomnia. Figure 1 represents the effects of the direct paths linking emotional exhaustion to each mediator and among mediators used in our research.

## Discussion

The aim of the present study was to test the relationship between emotional exhaustion and insomnia, considering the mediation of vigor, dedication and absorption as engagement factors. In our research, we found that emotional exhaustion was direct and significantly related to insomnia (*B* = 0.40; SE = 0.03; 95% CI = [0.33; 0.46]), confirming hypothesis 1. This is on the line of previous research which established strong direct relationships between these two variables [1, 2], Wand et al. 2020). Being exhausted at work and feeling overwhelmed by job demands is related to higher levels of insomnia. This relationship may impact not only in workers physical and mental health but also job performance. This may happen due to the perceived impairment of skills or reduced resources and increased fatigue levels because of exhaustion. Our research is on the line of those studies which have related burnout to insomnia [7], specifically, emotional exhaustion has been reported as a very important factor in predicting insomnia [15].

To achieve a more comprehensive optic, we decided to take engagement factors as mediators of the relationships between emotional exhaustion and insomnia. So, we tested the possible models to explain the complexity of the phenomenon. Our research is on the line with Leiter and Maslach [36] research where it was stated that burnout and engagement are different constructs. Based on this, we found that engagement factors have different roles in preventing insomnia.

Since the engagement concept has had many critics [26], our research has shown evidence in favor of engagement factors work differently on predicting insomnia. First, the most important factor in reducing the impact of emotional exhaustion on insomnia was vigor. We found enough evidence to confirm hypothesis 1a (B = 0.09, SE = 0.03; 95% CI = [0.03; 0.16]). As we mentioned above, vigor refers to the perception of high energy and mental resilience. Looking at our results, it seems clear that workers who felt exhausted when perceived high demands would need vigor to face work challenges. Following Job Demands and Resources Theory, vigor may act as a resource that helps to compensate for work demands. Within this optic, people who suffer high work demands, and perceive themselves as low on vigor, may be most likely to suffer insomnia.

When looking at the results of our models, model 1 (Exhaustion→ vigor→ insomnia) showed a statistically significant inverse relation to insomnia. When a worker feels vigorous, he can cope with difficulties and keep an alert state. However, when this vigor comes as a response to overcome exhaustion, this may buffer the exhaustion impact on insomnia. So, one of the stronger points of our research is to help to contextualize engagement and a better understanding of its processes. As previous research suggests, vigor is related to lower levels of insomnia, and may even work as a protective factor against this [37]. This may be explained because of the extra energy that a worker may feel to fulfill its work, even when exhausted. Future longitudinal research should evaluate if these relationships are maintained over time and the potential impact on health.

Going deeper into our model we found that the work engagement dedication factor does not show significant direct relationships with insomnia. So, we did not find enough evidence to confirm hypothesis 1b (B = −0.00; SE = 0.00; 95% CI = [−0.01; 0.00]). Dedication refers to the feeling of involvement in one’s job. It is related to concentration, and in our sample did not impact statistically significantly on insomnia. Some previous research stated that dedication appears to be the opposite of emotional exhaustion [38], and in our sample, the correlation showed the same pattern.

Regarding absorption, it showed an inverse correlation with insomnia. Besides, when introduced in the regression model with the other variables, it helped to buffer the impact of emotional exhaustion on insomnia. In our sample, we got enough evidence to confirm hypothesis 1c (B = 0.02; SE = 0.00; 95% CI = [0.01; 0.03]). Absorption is related to a state of full concentration on work, and positive engrossment in it [25]. It seems that, when taken on its own, it contributes to reduce insomnia probability, however, when one is absorbed in some tasks that may overwhelm his resources, this may impact on how a subject deal with his job. These relationships may occur because when workers feel exhausted, but concentrated in their jobs, they may try to fulfill their tasks. Workers may even invest more resources which may buffer the impact of exhaustion over sleep symptomatology. As noted above, future research should analyze these relationships over time to evaluate the evolution of these workers. This is a very interesting result because contributes to a deeper understanding of the organizational engagement processes.

Analyzing the models with two moderators in parallel, we found that model 4 (Exhaustion→ vigor→ dedication→ insomnia) was not significant in our sample (B = −0.04; SE = 0.03; 95% CI = [−0.10; 0.01]). It seems that emotional exhaustion follows other ways in its relationship with insomnia. So, in our sample, we did not find enough evidence to confirm hypothesis 2.

On the other hand, model 5 (Exhaustion→ vigor→ absorption→ insomnia) and 7 (Exhaustion→ vigor→ dedication→ absorption→ insomnia), achieve to revert the impact of emotional exhaustion over insomnia. However, model 6 (Exhaustion→ Dedication→ Absorption→ Insomnia) was not statistically significant. It seems that vigor is necessary to cope with job demands, but workers also need the capacity of being focused and involved in the task to cope with it successfully. This may be related to the active task coping strategies that help to solve the situation and decrease the demands of the workload. Our model may offer an interesting way of coping with emotional exhaustion demands on one’s job. Testing our hypothesis, we found enough evidence to confirm hypothesis 3 (B = −0.01; SE = 0.01; 95% CI = [−0.03; −0.10]) and hypothesis 5 (B = −0.026; SE = 0.01; 95% CI = [−0.05; −0.01]). It is very interesting that vigor is one of the most important components in our sample, and necessary to be considered to reduce exhaustion’s negative effects. Model 7 comprehends every component of engagement and helps to reduce exhaustion effects. When combined, engagement factors revert the effect of exhaustion on insomnia.

Proof of this is that when we eliminate vigor from the model, as we purposed in hypothesis 4, we did not find enough statistical significance to confirm our hypothesis (B = −0.00; SE = 0.00; 95% CI = [−0.01; 0.00]). Previous research stated that vigor was the opposite of feeling exhausted [39], but in our research, those workers who felt more vigorous tend to report fewer levels of insomnia.

On the other hand, our research has some limitations that should be addressed. First, the sample was acquired by not aleatory processes. This may conduct to a difficulty in representativity. Besides, our sample mostly was composed of individuals with higher education. This may impact the way that individuals cope with their jobs. Besides, another limitation is that our sample was composed of individuals with decision-making capacity (managers, middle-level management and administrative). This is a very important factor, because the degree of freedom in one’s job may impact how an individual face their job duties. Moreover, our research was conducted in only 1 time, and it should be necessary to follow individuals at different times, to check if the coping strategies are appropriate to reduce insomnia over time.

Future research should analyze if these peculiarities are maintained over time and evaluate the influence of the other burnout factors and its relationships to insomnia. Besides, future research should go deep into the relationships between the insomnia subtypes and burnout factors. Our research has a potential application in the organizational field because may offer managers the possibility to intervene in positions of responsibility, providing resources and freedom, to build healthier organizations. Furthermore, it would be interested to collect the number of participants who refuse to participate to gain more information about the sample and estimate the accuracy of the measures. Finally, future research should take work schedules into account. It may be expected that irregular work shifts may impact on insomnia symptoms.

## Conclusion

Our research offers an innovative way of understanding the nature and the job factors around insomnia. We can conclude that emotional exhaustion if no action is taken, is clearly and statistically related to insomnia. However, when engagement processes are included in the model, this relationship is buffered, showing even a negative relationship between exhaustion and insomnia. Despite vigor separably is the factor that showed a higher contribution to buffering the relationship between exhaustion and insomnia. Absorption tends to buffer the effect of emotional exhaustion over insomnia as well, but his impact is a little lower. Lastly, dedication is the less important factor buffering the effects of emotional exhaustion over insomnia being its impact negligible.
